# An update of the Worldwide Integrated Assessment (WIA) on systemic insecticides. Part 3: alternatives to systemic insecticides

**DOI:** 10.1007/s11356-017-1052-5

**Published:** 2018-02-25

**Authors:** Lorenzo Furlan, Alberto Pozzebon, Carlo Duso, Noa Simon-Delso, Francisco Sánchez-Bayo, Patrice A. Marchand, Filippo Codato, Maarten Bijleveld van Lexmond, Jean-Marc Bonmatin

**Affiliations:** 1 Veneto Agricoltura, Legnaro (PD), Italy; 2 Department of Agronomy, Food, Natural Resources, Animals and Environment, University of Padova, Viale dell’Università 16, 35020 Legnaro (PD), Italy; 3 Beekeeping Research and Information Centre, Louvain la Neuve, Belgium; 4 School of Life and Environmental Sciences, The University of Sydney, 1 Central Avenue, Eveleigh, NSW 2015 Australia; 5 Institut Technique de l’Agriculture Biologique (ITAB), 149 Rue de Bercy, 75595 Paris, France; 6 Condifesa Veneto, Associazione regionale dei ccnsorzi di difesa del Veneto, Via F.S. Orologio 6, 35129 Padova (PD), Italy; 7 Task Force on Systemic Pesticides, 46 Pertuis-du-Sault, 2000 Neuchâtel, Switzerland; 8 Centre de Biophysique Moléculaire, Centre National de la Recherche Scientifique (CNRS), Rue Charles Sadron, 45071 Orléans, France

**Keywords:** Systemic insecticides, Neonicotinoids, Fipronil, Mutual funds, Insurance cover, Pest control, Resistance, Biological control, IPM, Review

## Abstract

Over-reliance on pesticides for pest control is inflicting serious damage to the environmental services that underpin agricultural productivity. The widespread use of systemic insecticides, neonicotinoids, and the phenylpyrazole fipronil in particular is assessed here in terms of their actual use in pest management, effects on crop yields, and the development of pest resistance to these compounds in many crops after two decades of usage. Resistance can only be overcome in the longterm by implementing methods that are not exclusively based on synthetic pesticides. A diverse range of pest management tactics is already available, all of which can achieve efficient pest control below the economic injury level while maintaining the productivity of the crops. A novel insurance method against crop failure is shown here as an example of alternative methods that can protect farmer’s crops and their livelihoods without having to use insecticides. Finally, some concluding remarks about the need for a new framework for a truly sustainable agriculture that relies mainly on natural ecosystem services instead of chemicals are included; this reinforcing the previous WIA conclusions (van der Sluijs et al. Environ Sci Pollut Res 22:148-154, 2015).

## Introduction

After the publication of the World Integrated Assessment (WIA) on Systemic Insecticides (Bijleveld van Lexmond et al. 2015; van der Sluijs et al. 2015), some new research about neonicotinoids and fipronil have been made available. In this update, we have endeavored to collect all new information that has been published since 2014 onwards on the same topics covered by the WIA. The first review paper of the updated WIA (Giorio et al. 2017, this special issue) deals with the mode of action of neonicotinoids and fipronil, their metabolism, synergies with other pesticides or stressors, degradation products, and the contamination of the environment. The second updated WIA review covers the lethal and sublethal effects of neonicotinoids and fipronil on organisms, from aquatic and terrestrial invertebrates to vertebrates, and their impacts on ecosystems (Pisa et al. 2017, this special issue). The present review focuses on alternatives to the uses of these systemic insecticides for annual and perennial crops. Pest resistance to neonicotinoids and fipronil is also reviewed.

The prophylactic use of neonicotinoids and fipronil in crop protection is contrasted with integrated pest management (IPM) for controlling pests (Barzman et al. 2015; Furlan et al. 2016; Stenberg^2017^). We have divided this task in accordance with two main types of crops: (a) annual crops and (b) perennials (e.g., orchards and vineyards). A new Mutual Funds (MF) insurance approach that covers risk from IPM implementation, applied at a large scale for maize in Italy, showed that it is possible to bring numerous advantages for farmers and ecosystems when implementing IPM.

Abundant information is available about the negative impacts of neonicotinoids and fipronil on the environment, which are due to the sum of (1) their extreme toxicity to invertebrates (Pisa et al. 2015, 2017); (2) their high toxicity to vertebrates (Gibbons et al. 2015; Pisa et al. 2017); (3) their high persistence in soils and the contamination of surface water, both of which impact ecosystems and the services they provide (Bonmatin et al. 2015; Chagnon et al. 2015; Giorio et al. 2017; Pisa et al. 2017); and (4) their large-scale and widespread usage in all kinds of crops, even in non-agricultural settings (Simon-Delso et al. 2015; Douglas and Tooker 2015). However, there is a great deal of reluctance to reduce or phase out these insecticides because of fears that crops may experience yield losses and hurt farmer’s economies. Accurate information on the efficacy of prophylactic usage and other applications of these systemic insecticides and the environmental damage they cause should help resolve this issue in a rational way. Moreover, a suite of alternative methods already available, in the context of pest resistance to synthetic insecticides, should provide regulators with more sustainable possibilities for pest management of crops.

## Neonicotinoids and fipronil in agriculture

### Neonicotinoids and crop yields

Little information is available about the actual performance of neonicotinoids on crop production. However, concerns that crop yields might decrease significantly after the European moratorium (EU 2013a, b) of three neonicotinoids (clothianidin, imidacloprid, and thiamethoxam) and fipronil have been raised in the media and a few scientific publications (e.g., in Matyjaszczyket al. 2015 for maize and oilseed rape in Poland), although they were not supported by reliable data or statistics.

In Finland, yields of insect-pollinated crops are variable, whereas yields for wind-pollinated crops have been increasing for decades. While analyzing the possible factors related to yield declines for insect-pollinated crops, a significant linear correlation was found between the yield trends in rapeseed and the extent of neonicotinoid seed dressing used in provinces of that country (Hokkanen et al. 2017). In particular, yield declines in turnip rapeseed decreased as the use of neonicotinoid seed dressing increased. At the same time, the availability of honey bee colonies with respect to the growing area of crops benefitting from insect pollination had a linear, significant impact on turnip rapeseed yield trends. Since landscape and numbers of honey bee colonies had not changed during the period of the turnip rapeseed study, the authors of that study indicate that the only factor that could explain this decline in yields was the seed-treatment with neonicotinoids in the past 15 years.

In the UK, Budge et al. (2015) showed that yields of oilseed rape crops are not significantly increased by using imidacloprid in treated seeds. While the authors reported that farmers may get better economic returns some years, as they apply seed coatings which reduced the number of subsequent applications of foliar insecticide sprays, they also revealed a correlation over an 11-year-period between honey bee colony losses and national-scale imidacloprid usage patterns across England and Wales. These findings on oilseed rape yields are consistent with previous reports on the non-usefulness of neonicotinoids in soybean (Seagraves and Lundgren 2012) and wheat crops (Macfadyen et al. 2014).

In regard to maize, the available literature, mainly about studies in Italy, shows that the effect of seed-coated neonicotinoids on grain yield was mainly negligible (Furlan and Kreutzweiser 2015 citing different papers on field trials covering a 15-year period). This was mainly due to the fact that the majority of pest populations were under the economic injury level.

Other studies have shown that neonicotinoid insecticides can have effects on germination. For instance, Nogueira Soares et al. (2017) co-published with Syngenta that thiamethoxam improves physiological performances of melon and watermelon seeds treated with this neonicotinoid. By contrast, Tamindžić et al. (2016) have shown that three commercial formulations (i.e., Poncho, Gaucho and Cruiser) were harmful and reduced germination of three inbred maize varieties. The most harmful treatment was Gaucho (active ingredient (a.i.) imidacloprid) when compared to Cruiser (a.i. thiamethoxam) and Poncho (a.i. clothianidin, a derivative of thiamethoxam).

Deguines et al. (2014) analyzed a country-wide dataset of the 54 major crops in France produced over the past two decades. They found that the benefits of agricultural intensification decrease with increasing pollinator dependence, to the extent that intensification failed to increase the yield of pollinator-dependent crops and decreased the stability of their yield over time. The authors concluded that benefits from agricultural intensification may be offset by reductions in pollination services and support the need for an ecological intensification (reviewed by Kovács-Hostyánszki et al. 2017) of agriculture through optimization of ecosystem services. In other words, the prophylactic use of systemic insecticides, which impacts on both managed and wild pollinators (Pisa et al. 2015, 2017), is opposed to yield increases for pollinator dependent crops.

### Alternatives to systemic insecticides in agriculture

#### Annual crops

##### The use of systemic insecticides against key pests of annual crops

The use of neonicotinoids or fipronil against the main pests of annual crops was previously described in Furlan and Kreutzweiser (2015) and Simon-Delso et al. (2015). It was stressed that the main use of these systemic insecticides is prophylactic, for instance by means of seed coating techniques, and that this systematic approach is contrary to IPM principles (Furlan et al. 2016).

##### Alternative methods for pest control in annual crops

Some new IPM strategies that reliably limit the need for treatments involving neonicotinoids were also described in Furlan and Kreutzweiser (2015). The need for more data on the factors driving the risk of soil pest damage to maize and other susceptible crops, mainly to wireworm attacks, was emphasized in order to improve both the practical approach for farmers and the setup of specific MF to support IPM implementation. In practice, pest level evaluation is often not done mostly because of a lack of non-time-consuming and low cost methods (essential for low revenue crops) and insurance to cover for the risk of mistakes in pest population estimations. Some interesting recently published papers contribute to this issue: after a 29-year long-term study in Italy, the strongest factors increasing the risk of wireworm damage have been isolated, making now possible low-cost and reliable predictions to meet crop protection needs (Furlan et al. 2017).

A univariate analysis in the risk assessment was applied to identify the main factors that influence the occurrence of damage. Then, a multifactorial model was applied using the significant factors identified in the previous step. This model allows the strongest factors to be highlighted and to analyze how the main factors together influence the damage risk. The strongest factors were *Agriotes brevis* as the prevalent damaging species; organic matter content > 5%; rotation, including meadows and double crops; poor soil drainage; and *A. sordidus* as the prevalent damaging species. Also, the surrounding landscape with prevalent meadows was an important risk factor, confirming previous findings by other authors (Blackshaw and Hicks 2013; Benefer et al. 2012; Hermann et al. 2013; Saussure et al. 2015). The multifactorial model also showed how the simultaneous occurrence of two or more of the aforementioned risk factors can conspicuously increase the risk of wireworm damage to the maize crop, while the probability of damage for a field with no risk factors is always low (< 1%). These results make it possible to prepare risk maps for any country identifying low-risk and high-risk areas.

This information may be used to implement IPM and to tackle soil pests attacking maize in many European regions (Furlan et al. 2016) and beyond, which may lead to a considerable reduction in the use of soil insecticides and the immediate containment of the environmental impact of agriculture with no negative repercussions on farmers’ income. This can be achieved by implementing two phases: (i) “area-wide” risk assessment, including click-beetle population monitoring with pheromone traps (Furlan and Kreutzweiser 2015) and (ii) complementary field monitoring where risk assessment has identified the presence of risk factors (Furlan et al. 2016). When a harmful population is found, wireworm-activity-predicting models based on soil humidity and temperature may be useful to assess if the damage is really being done by larvae (Jung et al. 2014; Milosavljević et al. 2016).

The results of this work enable mapping of each cultivated region and high-risk areas to be pinpointed. Mapping the risk factors found in this survey, and that of Saussure et al. (2015) outside Italy, may allow us to prove that the cost–benefit of past soil-insecticide use was extremely negative. The first layer of the map includes the main soil characteristics (organic-matter content, texture, pH); the second includes the key agronomic characteristics (rotation, drainage); and the third the available entomological information, such as click-beetle population levels for the main *Agriotes* species, or wireworm presence/density assessed with bait traps over the years. A fourth layer reproduces the effects that occur when existing risk factors interact. This system enables areas with different risk levels to be highlighted. Each wireworm-risk category (e.g., low, medium, or high, based on the presence of one or more risk factors) will have its own IPM strategy, e.g., assessing wireworm density in high-risk areas or opting not to treat and not to continue monitoring in low-risk areas. Where risk factors are present, a precise procedure to spot land with an economic wireworm population has been described. In this way, control strategies will be implemented only when and where economic thresholds for maize are exceeded, and then it will be possible to avoid expensive soil insecticide use. Note that the risk factors causing high wireworm populations in maize are the same as those in non-maize crops. Therefore, they can be used to implement IPM in all arable crops, with possible adaptations.

Choosing fields with no risk factors may reduce the damage risk for all crops, including sensitive vegetable crops. Assessing the risk of wireworm damage affords a solid basis for estimating the amount of farmland that can be left untreated each season, without any risk of yield reduction. In Italy, implementing IPM is likely to result in a maximum of about 4% of maize-cultivated land being treated with soil insecticides or by using insecticide-coated seeds (Furlan et al. 2017). This means that 96% of these fields will not need any insecticide treatment. Precise IPM thresholds for soil pests in maize could be set everywhere. For instance, in no-risk areas, soil insecticides or insecticide-coated seeds may need to be used on no more than 1% of maize-cultivated land. In areas where organic matter content is over 5%, soil insecticides could be used on about 20% of maize-cultivated land if the prevalent species is *A. sordidus*. For large areas with scattered-risk situations, IPM thresholds will be a balanced mean of the damage risk caused by various risk factors and the surface area of cultivated land where each risk factor occurs. This could be immediately applied to areas harboring the species studied therein and to other areas shortly afterwards. In fact, local checks and adaptations should be assessed in regions where other species and/or conspicuous climatic differences occur, but the aforementioned IPM approach should be used since it is likely that the same main risk factors would play a key role. This would allow IPM to be extended wherever the *Agriotes* species studied in this work are widespread, and probably also to wherever other Elateridae species occur, once accurate comparisons have been made.

1. MF insurance cover

A low-cost IPM approach for low-risk pests based on risk factors and limited direct monitoring of fields makes it important that farmers get an appropriate compensation for the few fields that suffer from pest soil damage which differ from IPM predictions due to natural variability of the phenomena. In this case, risk insurance coverage may be extremely useful. Insurance cover/MF may be taken out privately by associated farmers, or with the support of EU regulations (Reg. 1305/2013/EU). With risks below 1%, a few Euros per hectare (about ten times less than soil-insecticide costs) would be enough to pay for damaged fields (Ferrari et al. 2015), including those damaged despite having been treated with soil insecticides, the likelihood of which is high (Saussure et al. 2015). MFs are instruments managed by collectives of farmers aimed at creating compensation through an interregional distribution of risks. They are non-profitable and have transparent rules. Compensation is commensurate with the financial resources of the Fund. The Fund stock is increased by savings in forecast costs. They cover risks that private insurance companies currently do not (e.g., climatic adversities such as flooding, and damage by wild animals and pests, just before and after the emergence of arable crops). The Italian Case study for implementation described here covered large-scale areas (> 47,000 ha) in two regions of high agricultural importance, Veneto and Friuli-Venezia Giulia.

In the above-cited regions, a long-term research and survey (> 29 years) demonstrated that economic damage risk by soil pests is less than 4%, and that a reliable IPM procedure is available to spot which fields really need protection from pests. As described above, the absence of risk factors greatly decreases the chance of economic damage and makes the application of soil insecticides in most fields useless. Where risk factors are present, an appropriate practice is to assess wireworm populations with bait traps and to introduce control strategies only when and where economic thresholds for maize are exceeded. This is clearly opposite to the systematic and prophylactic uses of systemic insecticides. Insurance covers also the risk of mistakes in IPM implementation, including any underestimation in the size of the area with wireworm economic populations (Furlan et al. 2015). Based on this risk assessment, a specific Maize Mutual Fund was set up. Its main features are summarized in Table 1. In essence, farmers should use pesticides only when they are really needed, based on IPM procedures suggested by the Annual Crops Bulletin (accessible at http://www.venetoagricoltura.org/subindex.php?IDSX=120), while getting insurance cover for mistakes and unexpected damages.

**Table 1:** Main features of the Mutual Fund (MF) strategy for maize in Italy.

Participants	Members of the farmer consortia
Obligations	• Contract to be signed within 7 days after sowing
	• Implementation of good cultivation practices
	• Implementation of Directive 128/2009/EC
	• Implementation of suggestions in the “*Annual Crops Bulletin*”
Risks covered	• Insufficient plant density (stand) due to adverse weather conditions (i.e., drought, flooding, freezing cold)
	• Insufficient plant density (stand) due to soil pests (e.g., wireworms, black cutworms)
	• Insufficient plant density (stand) due to diseases such as *Fusarium* spp. (rotten roots, seedlings)
	• *Diabrotica* (WCR) damage
	• Loss of production caused by wild fauna
Cost	€3–5/ha all inclusive (flooding, excessive rain, freezing cold, drought, pest risk, diseases, and wild fauna)
Compensation	• Changing crop for WCR damage (up to €1000/ha)
	• Up to €500/ha including the cost of:
	○ Re-sowing, if stand below 4 PLS/m^2^ (up to €250/ha)
	○Yield reduction because of sowing delay (up to €250/ha)

*PLS* pure life seed

The practical implementation of Maize MF in 2015–2016 resulted in the following economic and management effects: 47.558 ha were covered by the Maize MF on average over the 2 years; the cost was 3.3 €/ha (about one tenth the cost for a soil insecticide); total revenue to cover damage by wireworms, western corn rootworm, wild fauna, and other minor pitfalls was 160.335 €, while total damage paid was 83.863 € (~ 52%). Therefore, there was a very significant increase of the MF stock for following years.

The comparison between the prophylactic approach with soil insecticides to protect maize seeds/young plants at sowing and IPM approaches based on MF implementation are showed in Table 2. Costs of IPM implementation for farmers and of running MF are detailed below as farmer’s cost and institution’s cost:

1. Farmers’ costs of IPM implementation are given in the fifth column from left in Table 2. The obligation for farmers is to follow IPM suggestions of the Annual Crops Bulletin for an actual implementation of IPM principles. If just an evaluation of risk factors presence is done (see the fourth row from above), only 4–5 hours of a technician are needed (about 100€/100 ha). If a full IPM implementation is considered (see the third row from above), the estimated total cost is 1000€/100 ha. It corresponds to 16 ha at risk, as assuming they are monitored with bait traps (Furlan 2014). It includes 2 h/ha to do the monitoring—about 40€/ha with a subtotal cost of 640€, 60€ for materials, 100€ for travel costs and overheads, and about 200€ that might be needed for an accurate risk analysis with mapping of the cultivated fields and further insight. Note that in Europe, these costs should not be considered as additional costs since the compliance with IPM principles and the connection with IPM Bulletins are compulsory for all the crops in all Member States in Europe.
2. Institution’s costs are considered in the sixth column from left in Table 2. They correspond to a total MF cost of 5€/ha. This includes both 4€/ha of pure premium to cover the actual damage risk (a prudent figure higher than precise estimation done), and 1€/ha for specific administrative costs (including fixed costs) and for the costs of damage assessments by experts in fields where farmers ask to visit. Over the 4 years of practical implementation of MF, this latter cost per year ranged between 5 and 15% of the revenue from farmers to cover damage by pitfalls. More precisely, this maximum cost was 0.6 €/ha but, to follow a prudential approach, we overestimated this cost at 1 €/ha in Table 2.

**Table 2:** Comparisons of four agricultural strategies for maize in Italy

Strategy	Surface of reference (ha)	Surface with insecticide (ha)	Insecticide cost (€)	IPM cost (€)	MF cost (€)	Strategy cost (€)	Damage cost (€)	Total cost (€)	Differential cost^a^ (€)	Compliance with Directive 2009/128/CE	Risks for environment and public health	Synthetic evaluation scale (1 to 5)
MF (only)	100	0	0	0	500	500	2000	2500	-1500	Yes	None	+++++
IPM based on risk factors + monitoring + MF		10	400	1000	500	1900	500	2400	-1600	Yes	Low	++++
IPM based on risk factors + MF		20	800	100	500	1400	750	2150	-1850	Partial	Medium	+++
Soil insecticide (prophylactic)		100	4000	0	0	4000	0	4000	0	No	High	+

Data are based on four prudential assumptions for 100 ha of arable crops including 16% of land having the most important risk factors: (1) Mutual fund (MF) cost of €5/ha (worst case), (2) soil insecticide cost of €40/ha (realistic), (3) highest damage cost of €500/ha on 4 ha out of 100 (worst case), and(4) soil insecticide efficacy set at 100% (best case)

^a^Difference with respect to the soil insecticide strategy

Because of this generally low risk level, the crop insurance program (MF to protect maize at the early stages) proved to be more convenient than insecticide protection on large scale. Growers may purchase MF cover instead of soil insecticides, to provide financial compensation when yield losses can be attributed to pests or adverse weather conditions. In fact, the total cost of damage to maize (e.g., need for re-sowing and yield loss due to delayed sowing or reduced stand) plus the MF cost was much lower than the total cost of the soil insecticide treatments of most fields as the result of prophylactic protection approach (Table 2), even when all the fields are left untreated.

In the two intermediate IPM scenarios of Table 2, the assumptions were (1) a little bit larger land than that presenting risk factors is being treated, including all the border line cases, to minimize the risk of unpredicted damage; (2) since the efficiency of soil insecticide is set at 100% (an optimistic estimate), by enlarging the treated land the probability of finding a field with an economic damage is very low. In the practical application of these two IPM scenarios, economic damages observed in north-east Italy were < 0.1%, which is really negligible. However, to make the exercise extremely severe, we have considered in Table 2 a case worse than the worst case found in practice: a damage of 1 ha out of 100 ha (500€) is considered as unpredicted for IPM based on a risk factor evaluation and a monitoring scenario, whereas an unpredicted damage of 1.5 ha out of 100 (750€) was considered for the scenario without monitoring. Nevertheless, a great advantage of applying IPM instead of a prophylactic approach is clear. In addition to economic considerations, MFs avoid the environmental side effects of insecticides on beneficial species, biodiversity, ecosystems, and human health (Furlan et al. 2015; van der Sluijs et al. 2015; Cimino et al. 2017; Pisa et al. 2017).

When risks are low, the insurance approach is thus convenient for farmers and safe for people, biodiversity (including pollinators), the environment, and ecosystems. An insurance approach is much more cost-effective than insecticides since its large-scale and multiannual implementations demonstrated that MF costs are much cheaper for farmers than insecticide use. Obviously, the lower the damage risk is, the more efficient an MF becomes, even without any subsidy. The MF insurance approach can immediately reduce pesticide use and increase farmers’ net income by replacing pesticides with a lower cost strategy. Interestingly, MFs allow an increase of IPM application by making farmers more comfortable with IPM procedures, since mistakes in IPM implementation are also covered.

2. Biological control and natural derived insecticides

A few papers have recently been published on biological control and natural derived insecticides to control pests in arable crops. One new suggestion is about attract-and-kill strategies using biological tools against soil pests. Brandl et al. (2017) proved this strategy may reduce potato damage by wireworms in organic potato production systems in Lower Saxony, Germany. This strategy is based on the attraction of wireworms towards an artificial carbon dioxide-emitting source, using baker’s yeast (*Saccharomyces cerevisiae*) in combination with *Metarhizium brunneum* conidia for wireworm infection. This strategy offers the potential to promote biological wireworm control as an alternative to insecticide use by potentially reducing the inoculum compared to an inundate *M. brunneum* conidia release strategy (Kabaluk et al. 2007). This approach had some practical successful implementations in corn (Kabaluk and Ericsson 2007), like the use of biocidal plants and meals as described in Furlan and Kreutzweiser (2015). Kabaluk (2014) showed that applications of *M. brunneum* conidia may cause high mortality to adult *Agriotes obscurus* click beetles in field trials. This was followed by experiments that showed a potential for an attract-and-kill strategy also against *Agriotes* adults using sex pheromones (Kabaluk et al. 2015).

However, while biocidal plants and meals have become commercial products available for farmers, the setup of ordinary control tools based on the entomopathogens described above requires efforts for the future. A careful cost-benefit analysis is also needed to practically evaluate these alternative tools.

3. Ecological engineering for pest suppression: habitat manipulation for pest management and cultural control

In rice, ecological engineering practices that were first developed in China (Gurr et al. 2012) have been field tested in three countries: China, Thailand, and Vietnam, for multiple years (Gurr et al. 2016; Spangenberg et al. 2015). The results showed that in rice fields grown with flowers on the bunds, insecticide use was reduced by 70%, biological control was increased by 45%, pest populations were decreased by 30%, and yields were increased by 5%. These ecological engineering practices are now widespread in Vietnam (Heong et al. 2014) and China (Lu et al. 2015).

#### Perennial crops

##### The use of neonicotinoids against key pests of perennial crops

A large number of arthropod pests of temperate fruits (e.g., apple, pear, peach, and cherry) and grapevine have been managed for a long time using synthetic pesticides such as organophosphates and carbamates. Problems associated with their wide use, such as pest resistance, pest resurgence, and outbreaks of secondary pests, as well as concerns about their toxicity towards beneficial invertebrates and mammals, have progressively reduced their availability in many developed countries. Pyrethroids were suggested to replace these pesticides because of their relatively low toxicity towards mammals, but their impact on natural enemies of pests (i.e., predators and parasitoids) with consequent risks of secondary pest outbreaks reduced their appeal for growers involved in IPM in perennial crops (Duso et al. 2014). Additionally, similar risks for aquatic invertebrates as compared to neonicotinoids have been reported (Douglas and Tooker 2016). Chitin synthesis inhibitors were then successfully proposed due to their long persistence activity on target pests and relatively low acute toxicity to mammals. Later, their popularity also declined due to technical (e.g., pest resistance) and environmental issues (risks to aquatic crustaceans) associated with some active ingredients (Castro et al. 2012; Rebach and French 1996). More recently, neonicotinoids were proposed as a category of insecticides characterized by reduced risks to human health. A number of active ingredients showed a high efficacy in the control of sucking insects and other pests, probably because of their novel mode of action (MoA) and systemic distribution within plants (Bonmatin et al. 2015 and Giorio et al. 2017).

Aphids are key pests in apple and peach orchards. Pre-blossom pesticide applications are considered essential to reduce their damage to fruit production, and neonicotinoids proved to be effective in keeping these pests below economic thresholds in fruit orchards (Shearer and Frecon 2002; Beers et al. 2003; Lowery et al. 2005; Brück et al. 2009). In Europe, the use of three active ingredients (i.e., imidacloprid, thiamethoxam, and clothianidin) has been restricted to post-blossom applications due to their side effects on honeybees (EU Regulation 485/2013a, b; Pisa et al. 2015, 2017) and further restrictions have been applied in some countries. In some areas, the elimination of a number of broad-spectrum insecticides has been associated with an increase of the rosy apple aphids *Dysaphis plantaginea* Passerini (Cross et al. 1999; Solomon et al. 2000; Dib et al. 2016), thus prompting the use of neonicotinoids to control them. Neonicotinoids play also a role in the control of the San José scale *Diaspidiotus perniciosus* Comstock. An advantage of their use is the high systemic activity that allows an effective control of this pest (Buzzetti et al. 2015). In fruit orchards, other pests such as the codling moth, *Cydia pomonella* (L.), and the oriental fruit moth,*Grapholita molesta* (Busck), can be controlled with neonicotinoid applications (Jones et al. 2010; Magalhaes and Walgenbach 2011; Yang et al. 2016).

Neonicotinoids were suggested as an alternative tool in the control of medfly (*Ceratitis capitata* Wiedmann) and South American fruit fly *Anastrepha fraterculus* (Wiedemann) in peach and nectarine orchards (Raga and Sato 2011; Rahman and Broughton 2016). The results are not always successful, and other control measures, including attract-and-kill techniques, are recommended (Broughton and Rahman 2017).

The spotted wing drosophila, *Drosophila suzukii* Matsumura, is a serious pest of sweet cherry and other fruit crops. In trials carried out in North America, some neonicotinoids and OPs were effective against this pest (Beers et al. 2011), but in other trials, neonicotinoids seem less effective than other insecticides (Bruck et al. 2011; Shawer et al. 2018). Laboratory experiments suggested that acetamiprid can provide efficient control of *D. suzukii* when applications are performed before egg deposition (Pavlova et al. 2017). Wise et al. (2015) suggested that the use of neonicotinoids is not a good option in post-infestation applications.

Another pest of increasing importance worldwide is the brown marmorated stink bug (BMSB), *Halyomorpha halys* (Stål) (Hemiptera: Pentatomidae). Invasive in the USA and Europe, BMSB can attack various crops (Leskey et al. 2012). Against this pest, neonicotinoids are considered as an efficient option for its control (Kuhar and Kamminga 2017).

The Asian citrus psyllid *Diaphorina citri* Kuwayama (Hemiptera: Liviidae) is an economically important pest of citrus worldwide and the vector of the phloem-limited plant pathogen “Candidatus” *Liberi bacterasiaticus*, the presumptive causal agent of citrus greening disease (or huanglongbing). Neonicotinoid insecticides appear to be the most valuable option for containment of this pest (Ichinose et al. 2010).

Trunk injections of systemic insecticides (e.g., imidacloprid, acephate, dinotefuran) were tested to manage avocado thrips in California (Byrne et al. 2003). Acephate, which has been banned in the EU, was mobilized rapidly and proved to be effective against these thrips, but “unacceptable” pesticide residue contents were detected in fruits. Residues of imidacloprid and dinotefuran were significantly higher in leaves, whereas residues in fruits were below detection limits. The authors suggested that neonicotinoids may be a suitable control option against these pests.

In vineyards, neonicotinoids have been used for management of mealybugs (e.g., *Planococcus ficus* Signoret) (Wallingford et al. 2015). They were also used against ants that interact by mutualism with mealybugs and coccids in vineyards (Daane et al. 2008). Neonicotinoids, in particular imidacloprid, have been suggested against *Daktulosphaira vitifoliae* (Fitch) (Herbert et al. 2008), which is becoming aggressive again in some parts of Europe. These insecticides are effectively used to control leafhoppers (e.g., *Empoasca vitis* Goethe, *Erythroneura elegantula* Osborn, and *Scaphoideus titanus* Ball) in the vineyards of Europe and NorthAmerica (Van Timmeren et al. 2011; Žežlina et al. 2013). In the USA, drench application of neonicotinoids has been proposed against mealybugs and leafhoppers (Daane et al. 2008; Van Timmeren et al. 2011).

##### Alternative methods for pest control in perennial crops

1. Mating disruption

Mating disruption based on the use of synthetic sexual pheromones (Table 3) is an effective control tool against several pests, particularly tortricid moths such as *C. pomonella*, *G. molesta*, and *Lobesia botrana* (Den. and Schiff.) (e.g.,Witzgall et al. 2008; Ioriatti and Lucchi 2016). These methods can have some limitations in orchards and vineyards with uneven topography, high pest densities, and more generally in the first years after adoption. Successful control of these pests has been achieved using mating disruption with positive implications for insecticide use reduction and prevention of pesticide resistance (Trimble 1993; Angeli et al. 2007; Bohnenblust et al. 2011; Bosch et al. 2016; Calkins and Faust 2003; Ioriatti et al. 2011). However, recent research in Spain showed that the application of mating disruption in vineyards was associated with an increase of minor pest incidence (Gallardo et al. 2016). Pheromone-based mating disruption has also been developed against grapevine mealybugs with positive results (Walton et al. 2006; Cocco et al. 2014; Sharon et al. 2016). Mating disruption can be induced by substrate-borne vibrations that have been proposed in particular against some sucking pests that are known to use vibrational signals for communication during mating. Against these pests, vibration-based mating disruption tools have been tested showing potential for extensive applications (Polajnar et al. 2016).

2. Exclusion netting

**Table 3:** Summary of the main alternative methods in contrast with extensive, conventional, and intensive agriculture

Landscape	Farming methods	Organisms	Others
Patchy (reduced-size fields)	Mutual funds (insurance cover)	Macro-organisms:	Traps
Edge shrubs	Crop rotation	• Parasitoids	Attractants (traps)
Edge crops	Resistant variety:	• Predators:	Pheromones (traps)
Bund with flowers	• To insects	○ Vertebrates	Repellants
Wet zones (e.g., pond)	• To diseases	○ Invertebrates	Basic substances
Ecological corridors	Late sowing	Micro-organisms:	• Sugars
Trees (agroforestry)	Mixing varieties	• Fungi	• Oils
	Tillage	• Bacteria	• Nettle extracts
	Intercropping	• Nematodes	Mineral barrier (powders)
	Netting	• Viruses	Hot water (plant nursery)
	Stale seed bed		Sex confusion
	Removal of plants bearing pest		Chemical mediators
	Manual pruning		Plant defense stimulators
	Soil cover (e.g., grass)		Acoustic confusion
			Natural-derived insecticides

These methods are generally used in combination (without or) with low-risk pesticides for organic farming and IPM practices. These methods contrast with the prophylactic uses of highly toxic pesticides such as neonicotinoids and fipronil. Table adapted from Bonmatin (2016)

Exclusion netting with insect-proof screens represents another option for pest control (Table 3). Their use has been proposed to protect orchards from moth invasion, with positive effects in aphid population densities reduction (Dib et al. 2010; Sauphanor et al. 2012). More recently, the use of exclusion netting has been suggested for the control of the invasive pests *H. halys* and *D. suzukii* (Dobson et al. 2016; Rogers et al. 2016; Leach et al. 2016). These pests are generalist feeders that can repeatedly invade orchards during a season. Exclusion netting should provide a physical obstacle for the colonization of the crops. In addition, nets can be treated chemically, i.e., pyrethroid-impregnated nets. This tool could be used for the control of *H. halys* (Kuhar et al. 2017).

3. Biological control

Interest on the release of biocontrol agents to control pests in perennial cropping systems has increased in the last decades (Table 3). Inundative releases of egg parasitoids have been proposed against grape berry moths *L. botrana* (El Wakeil et al. 2008). However, it should be stressed that biological control not always ensures satisfactory levels of pest suppression and further research is needed to implement biological control strategies of tortricid moths in orchards and vineyards. Natural enemy releases have been proposed for the control of *Planococcus ficus* (Signoret) in vineyards of the USA, but the full efficacy of these tactics seem to be limited by climatic conditions (Daane et al. 2004).

The control of the woolly apple aphid *Eriosoma lanigerum* Haussmann represents a successful case of classical biological control by the hymenopteran parasitoid *Aphelinus mali* (Hald.). Outbreaks of the woolly aphids have often been associated with the negative effect of non-selective pesticides on parasitoid populations. Similar mechanisms can also be related to *Cacopsilla pyri* L. outbreaks in pear orchards (Solomon et al. 1989; Solomon et al. 2000; Vrancken et al. 2015). The role of predators in controlling peach aphids has been studied extensively (e.g., Pappas and Koveos 2011), but their real potential to keep pest populations under economic threshold levels needs further investigation. Recent research in aphid management showed that an increase in biological control can be indirectly obtained by excluding ants or by providing alternative sugar feeding to ants to reduce the ant-aphid mutualism (Nagy et al. 2013, 2015).

Within microbial pest control agents, a number of entomopathogenic fungi (e.g., *Beauveria bassiana* Bals. and *Paecilomyces fumosoroseus* (Wize)) have been evaluated for their activity against *M. persicae* and other aphid species with promising results (Andreev et al. 2012; Lefort et al. 2014; Lee et al. 2015). However, their effects in field conditions have been poorly explored.

Microbial products based on *C. pomonella* granulovirus (CpGV-M) have been suggested as an alternative in codling moth control (Cross et al. 1999; Beers et al. 2003), but these products have also been involved in resistance (Schmitt et al. 2013). Treatments with the entomophathogenic nematodes *Steinernema carpocapsae* (Weiser) and *Steinernema feltiae* Filipjev against overwintering larvae can achieve a good control of the codling moth (Unruh and Lacey 2001; Lacey et al. 2006) but their efficacy is strongly influenced by climatic conditions. Microbial control agents active towards tortricids and other lepidopteran pests include *Bacillus thuringiensis* Berliner (Cross et al. 1999; Lacey and Shapiro-Ilan 2008; Vassiliou 2011), characterized by its specificity towards Lepidoptera, Diptera, and Coleoptera and reduced risks to human health and the environment. The efficacy of *B. thuringiensis* can be limited by a number of climatic (e.g., temperature) and agronomic (e.g., differences in larval instar susceptibility, spray coverage, and application rate) factors that should be considered in practice.

4. Natural-derived insecticides

Besides biological control and mating disruption, alternatives in sucking pest control in perennial crops are represented by the application of natural-derived insecticides (Table 3). Multiple applications of kaolin, a clay mineral, in apple orchards have been found effective against a number of pests including the green apple aphid *Aphis pomi* (DeGeer) (Markó et al. 2008). In the same study, severe infestations of the woolly apple aphid have been found in kaolin-treated plots probably because of its negative impact on aphid natural enemies. The use of kaolin has been recently suggested against the grapevine leafhoppers in vineyards (Tacoli et al. 2017). Another option can be the use of fatty acid salts, in particular potassium salts, that have been used with a good efficacy against pear psylla (Souliotis and Moschos 2008).

Botanical insecticides such as those based on *Azadirachta indica* or natural pyrethrins have been suggested in controlling aphids in orchards and different other pests in vineyards (e.g., Andreev et al. 2012; Cichon et al. 2013; Dercks et al. 2014) but their effects were not always satisfactory. More recently, Annonaceae derivatives have been tested successfully in the laboratory against *M. persicae* (Ribeiro et al. 2014).

Among natural-derived pesticides, spinosad is a natural mixture of toxins produced by the soil actinomycete *Saccharopolyspora spinosa* (Mertz and Yao 1990). This compound proved to be effective to control codling moth, grape berry moths, leaf miners, thrips, and dipterans as a recurrent option in organic orchards and vineyards (Reissig 2003; Mota-Sánchez et al. 2008; Vassiliou 2011). However, its use has been associated with negative effects on natural enemies (e.g., predatory arthropods) of importance for fruit orchards and vineyards (Ahmad et al. 2013; Tirello et al. 2013; Duso et al. 2014; Pozzebon et al. 2014; Beers and Schmidt 2014; Malagnoux et al. 2015).

5. Food-derived biorationals as insect crop protection

More recently, natural or food-derived insecticides (e.g., vegetable oils), insect repellants (e.g., nettle extract), plant strengtheners (e.g., sucrose, fructose), and trap attractants (e.g., di ammonium phosphate) were approved as basic pest control substances under the European pesticide regulation, with no maximum residue limit (MRL) (Marchand 2015, 2016, 2017).

6. Ecological engineering for pest suppression: habitat manipulation for pest management and cultural control

Agro-ecological practices aimed at enhancing the effectiveness of natural enemies to reduce pest pressure can offer, for some perennial cropping systems, valid alternatives to insecticides in pest management.

In fruit orchards, peach aphid populations can be affected by chemical control measures but also by cultural practices and natural regulation. Fertilization has controversial effects on aphid dynamics and management. In an *ad hoc* experiment, *M. persicae* populations increased with a moderate number of treatments but decreased at higher number of treatments. The concentrations of primary and secondary metabolites in the plant were modified by the number of treatments, and this mechanism was suggested to be involved in these contrasting effects (Sauge et al. 2010). Four aphid control strategies, namely intensive, optimised, input-substitution, and integrated control, were compared in France (Penvern et al. 2010). The use of pesticides lowered densities of aphids as well as those of their natural enemies while cultural methods (e.g., ground cover and manual pruning of infested branches) promoted high populations of both arthropod groups. Data were critically discussed to redesign advanced orchard protection strategies that aimed to obtain pest control in the framework of biodiversity conservation. This approach requires local adaptations. For example, in China, the introduction of ground cover based on *Trifolium repens* L. in peach orchards obtained a significant reduction in the abundances of aphids and *G. molesta* (> 31%) probably due to the increase of generalist predators (> 115%) (Wan et al. 2014). The presence of natural enemies in pome fruit orchards can be promoted through habitat management practices such as increasing floral diversity in the agro-ecosystem by using selected trees and grasses (Rieux et al. 1999). The presence of hedgerows may increase the impact of parasitism of the codling moth (Maalouly et al. 2013; Monteiro et al. 2013).

In vineyards, the threats by sucking pests are mainly associated with leafhoppers (e.g., *E. vitis* and *E. elegantula*), mealybugs (e.g., *P. ficus*), thrips (e.g., *Drepanothrips reuteri* Uzel), and spider mites (*Panonychus ulmi* Koch and *Eotetranychus carpini* Oudemans). They are generally considered as secondary pests that can be managed by promoting the presence of natural enemies in vineyards (Duso et al. 2012, Walton et al. 2012). This can be achieved by increasing habitat complexity/diversity to provide refuges and alternative hosts and food resources to predators and parasitoids (Costello and Daane 2003; Duso et al. 2004; Ponti et al. 2005; Zanolli and Pavan 2011; Pozzebon et al. 2015a; Wilson et al. 2015) and reducing the use of non-selective pesticides (e.g., Jepsen et al. 2007, Pozzebon et al. 2015b). Biological control strategies against sucking pests can also be enhanced by inoculative or augmentative releases of natural enemies (Duso et al. 1985; Daane et al. 1996; Duso and Vettorazzo 1999; Daane et al. 2008). Irrigation, fertilization, and cultivar choice can be also managed to reduce pest incidence and economic damage (Daane and Williams 2003; Costello 2008; Fornasiero et al. 2012, 2016; Cocco et al. 2015).

### Resistance to neonicotinoids and fipronil

Since their commercialization in1991, neonicotinoids have been a useful tool for the control of various pests. The first case of resistance to neonicotinoids was reported in 1996, and later, a number of publications were devoted to this topic worldwide (Gorman et al. 2010). Increased use of insecticides exacerbates the development of resistance in most crop pests. In this regard, the use of neonicotinoids increased also rapidly after introduction of imidacloprid in several developed countries (Simon-Delso et al. 2015). The same situation has been observed since 2003 in the USA after the introduction of seed-treated crops in fields. The shift toward large-scale, prophylactic insecticide use was unprecedented, with 34–44% of soybeans and 79–100% of maize hectares treated in 2011 alone, contradicting previous expectations of using fewer insecticides than a decade or two ago (Douglas and Tooker 2015). One can expect, therefore, a rapid increase in pest resistance to all neonicotinoids in areas treated with coated seeds, as resistance mechanisms may develop rather quickly. For example, the cotton mealybug *Phenacoccus solenopsis* Tinsley (Hemiptera: Pseudococcidae) developed a 315-fold greater resistance to acetamiprid after five rounds of selection in controlled conditions, although at the cost of reducing the biological fitness of the resistant populations (Afzal et al. 2015). Other authors found that the development of resistance to acetamiprid in cotton fields takes 7 years and is slower than resistance to other neonicotinoids, carbamates, organophosphorus, and pyrethroid insecticides (Ahmad and Akhtar 2016).

#### Annual crops

Potential for resistance to neonicotinoids in the pests of arable crops has been described (Clements et al. 2017), and some cases of outbreaks of pest populations have been described in annual crops (e.g., in Santos et al. 2016 for soybean treated with imidacloprid in Brazil). More specifically, Santos et al. (2016) studied the survival and fertility of the Neotropical brown stink bug *Euschistus heros*. Newly emerged adult females were exposed for 48 h imidacloprid residues equivalent to 1% of the field rate dose. Females exhibited reduced rates of survival but higher fecundity and fertility rates compared with untreated females. The authors showed that females of *E. heros* increased their reproductive output in response to the imidacloprid sublethal exposure. These findings suggest a potential involvement of sublethal exposure to neonicotinoids in the recent outbreaks of the Neotropical brown stink bug *E. heros* observed in Brazilian soybean-producing regions.

In rice crops, resistance of the brown planthopper (*Nilaparvata lugens* Stål) to imidacloprid was first detected in Thailand in 2003, and then in Vietnam, Japan, and other Asian countries (Matsumura et al. 2008). Resistance of this pest to neonicotinoids is widespread in China, with resistance ratios (RRs, the greater it is, the higher resistance) in 2012 ranging from 209- to 617-fold. These values are much higher than in 2009. For thiamethoxam, the RR varied from 17 to 47 and for nitenpyram from 1.4 to 3.7 in 2012 (Zhang et al. 2014). Current levels of resistance are much higher than those reported 6 years earlier by Matsumura et al. in 2008. Similarly, populations of the whiteback planthopper *Sogatella furcifera* (Horvath) are resistant to fipronil in all countries of southeast Asia. In rice fields of Kumamoto, Japan, resistance of the small planthopper *Laodelphax striatellus* to fipronil has reached RR > 1,700, whereas new compounds with the same MoA (e.g., fluralaner acting on GABA- and l-glutamate-gated chloride channels) can be more effective in controlling this pest (Asahi et al. 2015). It seems that the cytochrome P450 CYP6ER1 is significantly overexpressed in imidacloprid-resistant planthopper populations of southeast Asia (i.e., *N. lugens* and *S. furcifera*), with higher tolerance levels of 10- to 90-fold compared with a laboratory-susceptible strain. However, other pest populations showed different overexpression of variant P450 enzymes implicated in imidacloprid resistance (Garrood et al. 2016). One study found that a single mutation at a conserved position (Y151S) in two nAChR subunits, Nla1 and Nla3, is responsible for a substantial reduction in specific imidacloprid binding (Liu et al. 2005). Even the additional use of neonicotinoid-specific synergists such as IPPA08 does not seem to work so efficiently with resistant populations of planthoppers (Bao et al. 2016). Therefore, the best option to control that pest would be IPM strategies that do not use neonicotinoids. Interestingly, the IPM strategy has been adopted in the Philippines. Results showed that populations of the above-mentioned planthopper in the Philippines remain susceptible to neonicotinoids (Matsumura et al. 2008) because of the many years implementing IPM with little use of insecticides in that country (Hadi et al. 2015). Actually, the obvious increase in resistance to systemic insecticides strongly suggests that management strategies other than chemical treatments are urgently needed to prevent damage by these pests.

In potato fields of North America, resistance of the Colorado potato beetle *Leptinotarsa decemlineata* (Say) to imidacloprid developed within 10 years after its introduction in 1995. By 2009, resistance affected more than 95% of the population of this pest in the Northeastern and Midwestern USA (Szendrei et al. 2012). High levels of resistance can be observed in areas within 100 km of the treated fields. It seems that the upregulation of three cytochrome P450s and a glutathione synthase-related protein provide a mechanistic explanation of resistance evolution in multiple resistant populations, but some of the resistant mechanisms involve also genetic changes and not just phenotypes (Clements et al. 2016). Resistance of this beetle species to another neonicotinoid, thiamethoxam, was first found in 2003 in a population from Massachusetts (Szendrei et al. 2012), further advocating that chemical control of this pest should be replaced with more rigorous application of IPM strategies.

In cotton crops, resistance of the tobacco thrips (*Frankliniella fusca* Hinds) to imidacloprid and thiamethoxam has developed faster than expected by earlier forecasts in southern areas of the USA. Some 57 and 65% of the populations monitored in 2015 showed resistance to the seed coated with neonicotinoids, with RR up to 55 and 39 for imidacloprid and thiamethoxam, respectively (Huseth et al. 2016). Resistance to thiamethoxam by the cotton aphid *Aphis gossypii* (Glover) has reached RRs between 29 and 526 in the USA (Gore et al. 2013), while in China, this pest shows a more moderate level of resistance to imidacloprid (RR of 42) and lesser values than for all other neonicotinoids (Shi et al. 2011).

The polyphagous and cosmopolitan whitefly *Bemisia tabaci* (Gennadius) is a devastating pest that can cause severe damage to a range of vegetable, fiber, and ornamental crops by direct feeding and by plant virus transmission. This species was the first to show resistance to imidacloprid and other neonicotinoids (Gorman et al. 2010). Cross-resistance among these compounds (Prabhaker et al. 2005) is threatening the chemical management program on genetically engineered cotton, where neonicotinoids are routinely being sprayed to manage sucking pests that have become dominant after the reduction in bollworms in the field (Basit et al. 2012).

The greenhouse whitefly *Trialeurodes vaporariorum* (Westwood) has also developed resistance to several neonicotinoids applied to vegetable crops in Europe and China (Table 4). The western flower thrip *Frankliniella occidentalis* (Pergande), which has invaded many horticultural and ornamental crops in China due to international trade, has developed up to 24-fold resistance to imidacloprid and up to 8.7-fold to acetamiprid (Wang et al. 2016a). This invasive pest also carries plant viruses that can decimate tomato and corn crops, so resistance to neonicotinoids strongly suggests that chemical control should be replaced with IPM strategies as well.

**Table 4:** Levels of resistance, illustrated by resistance ratios (RR), of common crop pests to neonicotinoid insecticides

Species/common name	Taxa	Chemical name	RR-fold	Crop	Country	Reference
*Amrasca devastans* (Distant) Cotton leaf hopper	Hemiptera: Delphacidae	Acetamiprid	2.3 to 29.3	Cotton	Pakistan	Saeed et al. (2017)
		Imidacloprid	4.8 to 95.0	Cotton	Pakistan	Saeed et al. (2017)
		Thiamethoxam	19.1 to 1197.9	Cotton	Pakistan	Saeed et al. (2017)
*Aphis gossypii* (Glover) Cotton aphid	Hemiptera: Aphididae	Acetamiprid	4.5	Cotton	China	Shi et al. (2011)
		Clothianidin	1.2	Cotton	China	Shi et al. (2011)
		Dinotefuran	1.1	Cotton	China	Shi et al. (2011)
		Imidacloprid	41.7	Cotton	China	Shi et al. (2011)
		Nitenpyram	5.8	Cotton	China	Shi et al. (2011)
		Thiacloprid	3.7	Cotton	China	Shi et al. (2011)
		Thiamethoxam	1.1	Cotton	China	Shi et al. (2011)
		Thiamethoxam	29 (3d) to 526 (2d)	Cotton	USA	Gore et al. (2013)
*Bemisia tabaci* (Gennadius) Silverleaf whitefly/sweetpotato whitefly	Hemiptera: Aleyrodidae	Acetamiprid	17 to > 2727	Cotton	Australia	Bingham et al. (2008)
		Acetamiprid	32 to 183	Melon, cotton, vegetables	USA (Arizona, California)	Castle and Prabhaker (2013)
		Acetamiprid	3.1	Cabbage	China	Liang et al. (2012)
		Acetamiprid	5 to 8	Cotton, melon	USA (Arizona, California)	Prabhaker et al. (2005)
		Acetamiprid	23	Melon	Guatemala	Prabhaker et al. (2005)
		Acetamiprid	36.6	Cotton	Pakistan	Basit et al. (2012)
		Dinotefuran	52 to 168	Melon, cotton, vegetables	USA (Arizona, California)	Castle and Prabhaker (2013)
		Imidacloprid	741 to > 2000	Cotton	Australia	Bingham et al. (2008)
		Imidacloprid	58 to 126	Cotton	Guatemala	Byrne et al. (2003)
		Imidacloprid	156 to 1830	Melon, cotton, vegetables	USA (Arizona, California)	Castle and Prabhaker (2013)
		Imidacloprid	6	Cotton	Egypt	Kady and Devine (2003)
		Imidacloprid	6	Cabbage	China	Liang et al. (2012)
		Imidacloprid	13 (larvae), 580 (adults)	Cabbage	Germany, Spain, UK	Nauen et al. (2008)
		Imidacloprid	120 to 160	Cotton, melon	USA (Arizona, California)	Prabhaker et al. (2005)
		Imidacloprid	109	Melon	Guatemala	Prabhaker et al. (2005)
		Imidacloprid	8.7 to 75	Cabbage	China	Yuan et al. (2012)
		Imidacloprid	7.25	Cotton	Pakistan	Basit et al. (2012)
		Nitenpyram	5	Cabbage	China	Liang et al. (2012)
		Nitenpyram	7.5 to 46.4	Cabbage	China	Yuan et al. (2012)
		Nitenpyram	28.4	Cotton	Pakistan	Basit et al. (2012)
		Thiacloprid	28.8	Cotton	Pakistan	Basit et al. (2012)
		Thiamethoxam	7 to 125	Melon, cotton, vegetables	USA (Arizona, California)	Castle and Prabhaker (2013)
		Thiamethoxam	2 to 22	Cotton, melon	USA (Arizona, California)	Prabhaker et al. (2005)
		Thiamethoxam	24	Melon	Guatemala	Prabhaker et al. (2005)
		Thiamethoxam	52.4	Cotton	Pakistan	Basit et al. (2012)
*Cimex lectularius* L Common bed bug	Hemiptera: Cimicidae	Acetamiprid	31.7 to > 33,000		USA (Ohio, Michigan)	Romero and Anderson (2016)
		Dinotefuran	46.8 to 358		USA (Ohio, Michigan)	Romero and Anderson (2016)
		Imidacloprid	2.0 to 462.6		USA (Ohio, Michigan)	Romero and Anderson (2016)
		Thiamethoxam	2.4 to 546		USA (Ohio, Michigan)	Romero and Anderson (2016)
*Cydia pomenella* Coddling moth	Lepidoptera: Tortricidae	Thiacloprid	5.5 to 16.5	Apple	Turkey	İşci and Ay (2017)
*Diaeretiella rapae* Parasitic wasp	Hymenoptera: Aphidiidae	Fipronil	Up to 20.8	Vegetables	China	Wu et al. (2007)
		Imidacloprid	Up to 74.7	Vegetables	China	Wu et al. (2007)
*Frankliniella fusca* (Hinds) Tobacco thrip	Thysanoptera: Thripidae	Imidacloprid	55	Cotton	USA	Huseth et al. (2016)
		Thiamethoxam	39	Cotton	USA	Huseth et al. (2016)
*Frankliniella occidentalis* (Pergande) Western flower thrip	Thysanoptera: Thripidae	Acetamiprid	Up to 8.7	Vegetables, roses	China	Wang et al. (2016a)
		Imidacloprid	2.8 to 24.4	Vegetables, roses	China	Wang et al. (2016a, b)
*Laodelphax striatellus* Small planthopper	Hemiptera: Delphacidae	Fipronil	> 1700	Rice	Japan	Asahi et al. (2015)
*Leptinotarsa decemlineata* (Say) Colorado potato beetle	Coleoptera: Chrysomelidae	Imidacloprid	1.8 to 27	Potato	USA (Wisconsin)	Clements et al. (2016)
		Imidacloprid	20 to 50	Potato	USA	Alyokhin et al. (2007)
		Thiamethoxam	6 to 9	Potato	USA	Alyokhin et al. (2007)
*Maconellicoccus hirsutus* (Green) Pink mealybug	Hemiptera: Pseudococcidae	Imidacloprid	10.2	Mulberry, vineyards	India	Mruthunjayaswamy et al. (2016)
*Musca domestica* L Housefly	Diptera: Muscidae	Thiamethoxam	6 to 76	Livestock farm	Denmark	Kristensen and Jespersen (2008)
*Myzus persicae* (Sulzer) Green peach aphid/peach-potato aphid	Hemiptera: Aphididae	Imidacloprid	Not yet	Orchards, beet root, grains	Australia	Edwards et al. (2008)
*Nilaparvata lugens* (Stål) Brown planthopper	Hemiptera: Delphacidae	Fipronil	3.7 to 5.4	Rice	Japan, Taiwan, Vietnam China	Matsumura et al. (2008)
		Imidacloprid	10 to 90	Rice	South East Asia	Garrood et al. (2016)
		Imidacloprid	3.5	Rice	India	Garrood et al. (2016)
		Imidacloprid	100	Rice	India	Gorman et al. (2008)
		Imidacloprid	Not resistant in 2006	Rice	China, India, Indonesia, Malaysia, Thailand and Vietnam	Gorman et al. (2008)
		Imidacloprid	22 to 89	Rice	Japan, Taiwan, Vietnam China	Matsumura et al. (2008)
		Imidacloprid	209 to 616	Rice	China	Zhang et al. (2014)
		Nitenpyram	1.4 to 3.7	Rice	China	Zhang et al. (2014)
		Thiamethoxam	2 to 3.4	Rice	Japan, Taiwan, Vietnam China	Matsumura et al. (2008)
		Thiamethoxam	17.4 to 47.1	Rice	China	Zhang et al. (2014)
*Phenacoccus solenopsis* (Tinsley) Cotton mealybug	Hemiptera: Pseudococcidae	Acetamiprid	9.7 to 315	Cotton	Pakistan	Afzal et al. (2015)
		Acetamiprid	Up to 30	Cotton	Pakistan	Ahmad and Akhtar (2016)
		Imidacloprid	7.6 to 217	Cotton	Pakistan	Afzal et al. (2015)
		Imidacloprid	Up to 151	Cotton	Pakistan	Ahmad and Akhtar (2016)
		Thiacloprid	Up to 338	Cotton	Pakistan	Ahmad and Akhtar (2016)
		Thiamethoxam	Up to 93	Cotton	Pakistan	Ahmad and Akhtar (2016)
*Sogatella furcifera* (Horváth) Whitebacked planthopper	Hemiptera: Delphacidae	Fipronil	Up to 37	Rice	Japan, Taiwan, Vietnam China	Matsumura et al. (2008)
		Imidacloprid	Up to 5	Rice	Japan, Taiwan, Vietnam China	Matsumura et al. (2008)
*Trialeurodes vaporariorum* (Westwood) Greenhouse whitefly	Hemiptera: Aleyrodidae	Acetamiprid	2.6 to 18.6	Vegetables	UK	Karatolos et al. (2010)
		Imidacloprid	2.6 to 21.8	Vegetables	UK	Karatolos et al. (2010)
		Imidacloprid	2.5	Eggplant	China	Liang et al. (2012)
		Nitenpyram	3.7	Eggplant	China	Liang et al. (2012)
		Thiamethoxam	1.3 to 20.4	Vegetables	UK	Karatolos et al. (2010)

Resistance of the bed bug parasite is also included

#### Perennial crops

Bass et al. (2015) reviewed most of the literature on pest resistance to neonicotinoids. Among pests of interest for perennial cropping systems, the green peach aphid *Myzus persicae* Sulzer has been involved in the highest number of reported cases of resistance among fruit trees. This appears to be a case of pre-selection resulting from host-plant adaptation (tolerance to nicotine by feeding on tobacco) and an expansion in host range. Resistance seems to be associated with metabolic detoxification by enhanced expression of cytochrome P450s. In some cases, it has also been found that modified penetration through the cuticle might contribute to resistance together with enhanced detoxification (Puinean et al. 2010). Target site resistance was also suggested as a mechanism inducing resistance in *M. persicae* (Bass et al. 2015). This was found to be associated to the R81T mutation in nAChR subunit genes in aphid populations from peach. Toda et al. (2017) developed a molecular diagnosis test for detecting the R81T mutation on the D-loop region of the β1 subunit of the nAChR gene; this mutation confers resistance to neonicotinoids in the cotton aphid *Aphis gossypii* (Hemiptera: Aphididae). This mutation appears to be distributed in aphid populations on peach and closely related crops over southern Spain, southern France, and northern and central Italy and Greece (Bass et al. 2015; Voudouris et al. 2016). Bass et al. (2015) stressed the need to employ insecticides with different MoAs to reduce the selection pressure induced by neonicotinoids.

In orchards, *M. persicae* has become resistant to imidacloprid and thiacloprid in populations of southern Europe. In Italy, 65% of the aphids studied by Panini et al. (2014) had the neonicotinoid-specific R81T mutation while a few genotypes also revealed the involvement of P450-based metabolic resistance processes (Panini et al. 2014). The dominance level of insecticide resistance in this species suggests that the mutant allele 81T is semi-recessive, with the wild 81R allele being rather dominant (Mottet et al. 2016). The neonicotinoid sulfoxaflor, a newly developed agonist of the nicotinic receptors (Giorio et al. 2017), appears to behave in a similar way in resistant strains of the same aphid *M. persicae* (Cutler et al. 2013), although some authors say is not affected by this mutation (Wang et al. 2016b). In populations of this aphid in Greece, over 58% of the clones collected in 2013 showed a 9- to 36-fold overexpression of the CYP6CY3 gene that codifies for the P450 detoxification mechanism (Voudouris et al. 2016). This is a matter of concern as tolerance can be developed very quickly by this mechanism.

The most important pest of apple trees, the codling moth *Cydia pomonella*, has developed resistance to thiacloprid, with RRs between 5.5 and 16.5 measured in orchards of Turkey (İşci and Ay 2017). Resistance of this pest to thiacloprid is correlated with mixed-function oxidase activity (Reyes et al. 2007). This phenomenon seems to be spread across the world (Bass et al. 2015; İşci and Ay 2017) and is linked to cross-resistance to other compounds such as organophosphates: in this case, the resistance mechanism seems to be based on detoxification enzymes. In other studies on *C. pomonella*, a number of detoxification genes (CYP9A61, CpGST1, and CpCE-1) were differentially induced or suppressed by various insecticides (including imidacloprid) while expression of these genes was not influenced by acetamiprid when compared to the control (Yang et al. 2016).

Other authors have shown that over transcription of a single gene product, Cyp6g1, which is associated with the metabolic resistance to neonicotinoids in *Drosophila melanogaster* larvae, results in a significant increase of three imidacloprid metabolites *in vivo* (Joussen et al. 2008; Hoi et al. 2014). The high frequency of mutations and data obtained from these studies confirm the existence of multiple resistance mechanisms (e.g., enhanced detoxification, mutations, overexpression of enzymes), which may require different management strategies.

On the Asian citrus psyllid *D. citri*, a reduced sensitivity to neonicotinoids in certain populations of this pest in Florida has been found, raising concerns that resistance to neonicotinoids can hamper management of this pest (Tiwari et al. 2011). The promotion of effective rotations of insecticides and area-wide management of *D. citri* seems to have determined a reversal for insecticide resistance in this pest (Coy et al. 2016).

Western flower thrips, *Frankliniella occidentalis* (Pergande), is a generalist pest that can threaten fruit orchards and vineyards. Metabolic resistance to neonicotinoids has been reported for this pest, probably originating by cross-resistance with other insecticides (Zhao et al. 1995; Minakuchi et al. 2013). In vineyards and mulberry groves of India, resistance of the pink mealybug (*Maconellicoccus hirsutus* Green) to imidacloprid have reached RR of 10.2-fold, similar to the tolerance found with other insecticidal classes (Mruthunjayaswamy et al. 2016).

Populations of the tea green leafhopper *Empoasca vitis* have been developing resistance to a number of insecticides in southeastern China, with high levels of cross resistance among imidacloprid, chlorfenapyr, and indoxacarb (Wei et al. 2017).

Resistance to neonicotinoids and fipronil has also been observed in beneficial insects. For instance, the parasitic wasp *Diaeretiella rapae* (Hymenoptera: Aphidiidae) showed cross-resistance to fipronil and imidacloprid and is now 20 and 75 times respectively more resistant to these chemicals than in the past (Wu et al. 2004).

Indeed, among the most common pests of agricultural and ornamental crops, resistance to systemic insecticides is now widespread and develops quickly, as it typically involves enhanced detoxification by GST and P450 enzymatic systems. In the case of neonicotinoids, specific mutations of the α-subunit of nAChRs confer long-term resistance to all chemicals of this class (Thany 2010). Highest resistance levels were found for imidacloprid, the first neonicotinoid launched to the market and lowest in the newest compounds like dinotefuran and nitenpyram (Shi et al. 2011; Zhang et al. 2014)—see Table 4. While resistance due to detoxification mechanisms can be overcome by using synergistic mixtures with other chemicals (Bingham et al. 2008; Basit et al. 2013; Darriet and Chandre 2013), the mutant-resistant individuals could be selected naturally rather quickly and eventually dominate the field populations of pests.

#### Other resistance to neonicotinoids

Pest resistance to neonicotinoids has been found not only in crops but also in the control of bed bugs (*Cimex lectularius* L.) in the USA. Consequently, formulations combining two neonicotinoids or a pyrethroid are currently becoming very popular in that country. However, high levels of resistance to four neonicotinoids, acetamiprid (up to RR of 33,000), imidacloprid (RR in the 2–463 range), dinotefuran (RR in the 47–359 range), and thiamethoxam (RR in the 2.4–546 range) have already been detected in bed bug populations. In this case, detoxification mechanisms by induction of glutathione S-transferases (GST) and cytochrome P450s are responsible for the development of such resistance, thus limiting the options for chemical control of bed bugs (Romero and Anderson 2016).

## Concluding remarks

Insecticides are expected to achieve higher yields and net incomes, but the relationship between yields and farmer’s profits is not so obvious. For example, the effect of insecticides on yield may be negligible (see examples above), or quality products under organic/“integrated” cultivations may be sold at higher prices than conventional ones treated with insecticides, thus largely compensating reduced yields. An example at the scale of west and east Germany is given by Batáry et al. (2017). Another large-scale example is given here with mutual funds and IPM, which increased farmer profits while reducing the use of pesticides without negative impact on average yields and at the same time avoiding environmental impacts.

A review of the current literature on neonicotinoids and fipronil shows these systemic insecticides have a role in protecting certain crops against the damaging attacks of some soil pest such as wireworms and root worms and of sucking pests, in particular aphids, leafhoppers, thrips, mealybugs, and scale insects, as well as internal grubs that can only be reached by chemicals translocated within the plant. However, their efficacy does not guarantee an increase of yield of the crops they are protecting, particularly in pollinated crops. This is not unusual, as a recent study in France demonstrated that insecticide usage hardly accounts for any yield benefit in arable crops (Lechenet et al. 2017), mostly because plants compensate for the small damage that insects inflict them while the risk of a pest outbreak is small on a year-to-year basis.

Pest management can be implemented effectively by using the multi-faceted methods of IPM described succinctly in this paper. In addition, economic insurance initiatives, as described for the case of maize crops in Italy, can make up for farmers’ losses in bad years, and they do not place any pressure on the environment, whereas neonicotinoids and fipronil do have large impacts on biodiversity, ecosystems, and ecosystem services worldwide (Pisa et al. 2017). In this sense, Europe is committed to continuing agricultural production while reducing significantly the amount of pesticide uses (Lescourret 2017) by making compulsory the use of IPM practices aimed at the preservation of environment and ecosystem services that sustain agricultural productivity (Sgolastra et al. 2017). Other countries (e.g., Canada) have taken regulatory decisions to reduce both non-agricultural and agricultural uses of neonicotinoids.

Here, we highlighted that the use of neonicotinoids is limited by the rapid development of resistance in target pests. Because many of the underlying mechanisms of resistance are common to other insecticide classes (e.g., pyrethroids, cholinesterase, inhibitors), the use of new neonicotinoids (e.g., sulfoxaflor, flupyradifurone; see Pisa et al. 2017) or substances with the same MoA is not the solution in the medium and longterm. It can even worsen impacts on non-target invertebrates by potential synergistic interactions with other neonicotinoids which are now everywhere in the environment (Mitchell et al. 2017). As Barzman et al. (2015) have said, “The future of crop production is now also threatened by emergence of pest resistance and declining availability of active substances. There is therefore a need to design cropping systems less dependent on synthetic pesticides*.*” Moreover, it is the prophylactic uses of such systemic insecticides in seed treatments that should be urgently stopped since they are contrary to IPM practices. The tools for a new cropping system that does not rely on chemicals alone have been with us for years, but the implementation of the IPM practices is lacking (Hokkanen 2015) despite the initial aspirations of the EU directive establishing a framework for community action to achieve the sustainable use of pesticides (EU 2009).

In the meantime, the overwhelming evidence of negative effects on pollinators and arthropods needs to be weighed against the pest control benefits that these systemic insecticides are supposed to produce (Chagnon et al. 2015). Over-reliance on chemical control is associated with contamination of ecosystems (Bonmatin et al. 2015; Pisa et al. 2015; Mineau and Whiteside 2013; Beketov et al. 2013; Giorio et al. 2017) and undesirable health effects (Scott et al. 2014; Cimino et al. 2017; Wang et al. 2018), although in the case of neonicotinoids and fipronil, the scarcity of studies on human health to date preclude us from making a clear assessment. More effort is needed to investigate the effects induced by these agonists of the neuronal system after chronic human exposure (e.g., farmers and workers, exposure by drinks, food, treated pets and breeding animals, treated wood structures, air pollution, etc., and the sum of all these exposures) (Salis et al. 2017).

We hope that this review may help regulators to carefully consider the pros and cons of the continuous, increasing, and widespread use of these systemic insecticides. On a scientific basis, the efficiency of neonicotinoids and fipronil for pest control should be balanced against the drawbacks of their damage to natural enemies and other ecosystem services that sustain agricultural systems.

We have restricted this WIA to neonicotinoids and fipronil because they represent most of the insecticide market nowadays. However, regulators should consider that replacing one molecule by another in the future is not a sustainable strategy for agricultural production, as new molecules with the same MoA (e.g., sulfoxaflor, flupyradifurone) are additional threats to the environment and public health. Regulators should realize that a more restrictive regulatory framework is required for more sustainable agricultural practices such as IPM, with a strong willingness to use (present or future) highly toxic pesticides only as the last resort.
